# Solution of Shellac

**Published:** 1853-01

**Authors:** 


*Solution of Shellac.*—The costliness of the solution of gun cotton and of gutta percha renders it desirable to have a cheaper article that may be used as a substitute in cases which require the consumption of a large quantity of such plastic materials. A solution of shellac in alcohol has therefore been proposed for this purpose. This may be prepared by adding successively small bits of shellac to the alcohol of commerce until enough be dissolved to make a mucilaginous solution.

Some of the French practitioners having attributed to collodion extraordinary antiphlogistic properties when applied over affected joints and other inflammatory affections, even more deeply seated, I determined to try, during the last winter, the shellac solution in an old case of rheumatism, in which most of the joints of the extremities were being successively invaded. The toes, ankles, knees, fingers, wrist and elbows were nearly all alternately implicated—becoming very painful and rapidly swelling, so as to be almost doubled in size in a day or two. I furnished the patient a bottle of the shellac solution and ordered it be painted over and around the joint as soon as it would commence to be painful, and to repeat the application several times a day until a thick coating remained, after which it might be applied only once a day. Under this treatment I was gratified to find that the patient could, in a few hours, arrest the pain and prevent the swelling of the joints to which he made the application. He stated that he never had anything to give him such prompt and effectual relief, although he had been suffering such attacks every winter for the last ten years. One joint or another continued to annoy him for a month, during all of which time he resorted to the shellac with the same success.

This is the only case in which I have tried this solution.—*Ibid.*

